# Design and Efficacy of Oncolytic Viruses and Antitumor Vaccines: A Dead End in the Immunotherapy of Pancreatic Cancer?

**DOI:** 10.3390/ijms26199640

**Published:** 2025-10-02

**Authors:** Eduard Achim, Elena Pîrlici, Cecilia Cristea, Mihaela Tertis

**Affiliations:** 1 Analytical Chemistry Department, Faculty of Pharmacy, “Iuliu Haţieganu” University of Medicine and Pharmacy, 4 Louis Pasteur St., 400349 Cluj-Napoca, Romania; achim.eduard.marian@elearn.umfcluj.ro (E.A.); ccristea@umfcluj.ro (C.C.); 2 Faculty of Medicine, “Iuliu Hațieganu” University of Medicine and Pharmacy, 400012 Cluj-Napoca, Romania; pirlici.elena.maria@elearn.umfcluj.ro

**Keywords:** oncolytic viruses, antitumor vaccines, pancreatic cancer, immunotherapy

## Abstract

Pancreatic ductal adenocarcinoma (PDAC) remains one of the deadliest malignancies, marked by late diagnosis, limited responsiveness to conventional therapies, and an immunosuppressive tumor microenvironment. While immunotherapy has transformed treatment paradigms in several cancers, its efficacy in PDAC has been minimal. Oncolytic viruses and therapeutic cancer vaccines have emerged as promising immunotherapeutic strategies designed to stimulate robust, tumor-specific immune responses and reshape the immune landscape. However, despite encouraging preclinical data, clinical translation in PDAC has been largely disappointing. This review critically evaluates the design, delivery, and efficacy of oncolytic virotherapy and cancer vaccines in PDAC, examining barriers such as stromal desmoplasia, immune exclusion, and tumor heterogeneity. We also explore combination strategies integrating checkpoint inhibitors, chemotherapy, radiotherapy, and stromal modulation to overcome resistance. Ultimately, the viability of these approaches hinges on a clearer understanding of their mechanistic limitations and the refinement of delivery platforms. These factors will determine whether oncolytic viruses and cancer vaccines can be successfully repositioned within the therapeutic arsenal or warrant reevaluation in the evolving landscape of PDAC treatment.

## 1. Introduction

Pancreatic cancer is among the deadliest malignancies and is projected to become the second-leading cause of cancer-related deaths in the United States [1]. Risk factors for Pancreatic Ductal Adenocarcinoma (PDAC), such as smoking, alcohol use, chronic pancreatitis, obesity, and processed meat consumption, are linked to its rising incidence, particularly in younger individuals [2].

Despite treatment advancements, prognosis remains poor. Only 10–15% of patients are diagnosed with resectable disease, with a 5-year survival rate up to 37.4%. However, 30–35% present with unresectable but localized tumors, and over half are diagnosed with advanced metastatic disease, where survival drops to 12.4% for regional and just 2.9% for metastatic stages [2,3]. According to SEER (Surveillance, Epidemiology, and End Results Program) data, the five-year survival rate for pancreatic cancer is approximately 44% for localized disease, 16% for regional disease, and 3% for distant metastatic disease [4].

**Figure 1:**
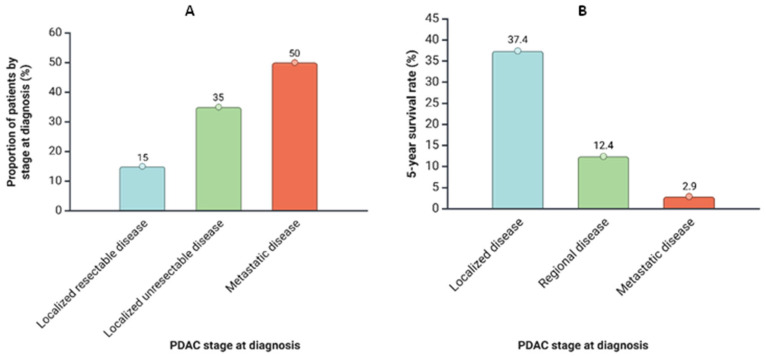
(**A**) Distribution of patients diagnosed with PDAC by stage at diagnosis; (**B**). Distribution of the 5-year survival rate for patients diagnosed with PDAC according to the stage at diagnosis.

The statistical data presented here are graphically represented in Figure 1.

The tumor microenvironment (TME) is a major contributor to therapy resistance. Characterized by dense desmoplastic stroma, it impedes drug penetration and immune cell infiltration while promoting hypoxia and nutrient deprivation. This drives metabolic reprogramming and tumor survival [5]. Additionally, the TME fosters immunosuppression through regulatory T-cells (Tregs), myeloid-derived suppressor cells, tumor-associated macrophages (TAMs), and cancer-associated fibroblasts (CAFs) [6].

Recent findings suggest PDAC progression may be more rapid than previously believed, driven by early, non-gradual mutations in KRAS, CDKN2A, TP53, and SMAD4, contributing to genomic instability and treatment resistance [7].

Surgery remains the most effective intervention, yet feasible for few. The actual 5-year survival remains below 5%, highlighting the need for earlier detection and more effective treatments [8]. FOLFIRINOX and gemcitabine/nab-paclitaxel are current standards, though radiotherapy’s benefit is still debated [9].

Immunotherapy has had limited impact, though targeted treatments show promise. Pembrolizumab is approved for MSI-H/dMMR tumors (KEYNOTE-158) [10], and olaparib for BRCA-mutated metastatic PDAC (POLO trial) [11].

Besides oncolytic viruses and cancer vaccines, other innovative therapeutic strategies for PDAC include antibody-based and cell-based strategies. Recent reviews have highlighted the major progress of antibody therapeutics in oncology [12], yet their clinical efficacy in PDAC remains limited, due to its dense stroma, immunosuppressive environment, and poor tumor infiltration. By contrast, oncolytic viruses and cancer vaccines are particularly promising because they can both kill tumor cells and prime antitumor immunity, which is why this review focuses on these modalities.

Given these limitations, novel strategies like oncolytic viruses and antitumor vaccines are under exploration for their potential to enhance therapeutic efficacy in PDAC.

## 2. Oncolytic Viruses in PDAC

Oncolytic viruses are immunotherapeutic agents that selectively infect and lyse tumor cells while stimulating systemic antitumor immunity through the release of tumor-associated antigens (TAAs). Despite promising preclinical data and encouraging clinical trials, key challenges persist, including poor tumor penetration, pre-existing immunity (e.g., to HSV), antiviral responses, and limited monotherapy efficacy [13]. Oncolytic viruses are classified by genetic material: DNA viruses (e.g., adenovirus, Vaccinia virus (VACV), Herpes simplex virus (HSV)) and RNA viruses (e.g., reovirus, measles virus (MeV), Newcastle disease virus (NDV), influenza) [14]. Ongoing research focuses on optimizing delivery, enhancing immune activation, and combining these agents with other therapies to improve outcomes in pancreatic cancer and other cancers.

### 2.1. Adenoviruses

Adenoviruses are among the most extensively studied vectors in oncolytic virotherapy. These non-enveloped, double-stranded DNA viruses include over 100 serotypes across seven species (A–G). Serotypes 2 and 5 of species C are frequently used in gene therapy due to their efficient gene delivery. Although adenovirus infections are generally mild in healthy individuals, they can cause severe illness in immunocompromised patients [15]. The adenoviral genome comprises early (E1–E4) and late (L1–L5) regions. Early genes initiate replication, while late genes encode structural components [16]. In infectious disease vaccines, adenoviruses are often rendered replication-deficient by deleting E1A. In cancer therapy, however, replication is essential for inducing tumor cell lysis. Conditionally replicating adenoviruses (CRAds) exploit dysregulated pathways in cancer cells to achieve selective replication [17]. One strategy for tumor specificity involves placing E1A under a tumor-specific promoter, restricting replication to malignant cells. Another leverages retinoblastoma pathway defects, a hallmark of many tumors. Normally, pRb inhibits E2F, blocking cell cycle progression. Adenoviruses use E1A to bind pRb, releasing E2F and enabling replication [18]. The Δ24 mutation in E1A prevents this interaction in normal cells, thereby increasing tumor selectivity, where pRb is frequently dysfunctional and the E1A deletion becomes functionally redundant [19].

Genetically enhanced CRAds have shown improved potency. LOAd703, incorporating Δ24 and expressing trimerized CD40L, stimulates immune activation [20]. VCN-01, derived from Ad5-Δ24, expresses hyaluronidase to degrade tumor stroma and enhance viral spread [21]. ONYX-015, with deleted E1B55K gene, selectively replicates in p53-deficient tumors [22].

Clinical trials show varying results. Early studies with ONYX-015 in pancreatic cancer patients demonstrated safety but limited efficacy, with inconsistent viral replication and modest disease control [23,24]. In contrast, newer CRAds offer greater potential. LOAd703 has shown enhanced antitumor activity in preclinical PDAC models and is currently under clinical evaluation with gemcitabine and nab-paclitaxel [25]. VCN-01 has shown selective tumor targeting, stromal breakdown, and early clinical benefit in combination regimens for PDAC [26].

These advances underscore the promise of engineered adenoviruses as oncolytic agents, particularly when paired with chemotherapy or immune modulators, offering a more precise and potentially effective approach to treating PDAC.

### 2.2. Measles Virus

MeV is a promising oncolytic agent due to its natural cytolytic properties and capacity to stimulate antitumor immunity. Its surface glycoproteins, the hemagglutinin and fusion proteins, facilitate host cell entry, with the H protein binding cellular receptors and the F protein mediating membrane fusion. Wild-type MeV uses the CD150 (SLAM) receptor to infect immune cells, aiding systemic spread, and the nectin-4 receptor for epithelial infection. Nectin-4 is often overexpressed in tumors, enabling selective targeting in cancer therapy [27,28].

Attenuated vaccine strains utilize CD46, a receptor broadly expressed on human cells except erythrocytes. Syncytia formation, a hallmark of MeV infection, requires high CD46 expression, common in tumors but not in normal tissues, enhancing tumor selectivity [29]. Additionally, many tumor cells have impaired interferon (IFN) signaling, weakening antiviral defenses and facilitating MeV replication [30]. Tumor-driven oncogene expression further suppresses IFN-β, contributing to immune evasion [31].

To refine tumor specificity, MeV has been genetically modified. H protein engineering reduces affinity for normal receptors while targeting tumor-specific antigens. Additionally, insertion of microRNA (miRNA) target sites into the viral genome limits replication in normal cells where these miRNAs are expressed, exploiting their downregulation in tumors [32].

MeV has also been armed with therapeutic transgenes to enhance cytotoxicity or stimulate immunity, including purine nucleoside phosphorylase (PNP), super cytosine deaminase (SCD), immunostimulatory cytokines (GM-CSF, IFN-β), TAAs, BiTEs, and anti-angiogenic factors.

Combination therapies are gaining traction. Histone deacetylase inhibitors (HDACis), like resminostat, enhance MeV efficacy by suppressing IFN-stimulated genes and promoting autophagy [33]. JAK inhibitors such as ruxolitinib also amplify viral oncolysis by further dampening antiviral responses [34]. In PDAC models, resminostat combined with MeV increased tumor cell death beyond either agent alone, without affecting viral replication, suggesting synergy via epigenetic modulation [35].

Gemcitabine-MeV combinations have demonstrated strong synergy, reducing tumor cell viability below 30%, outperforming either monotherapy, and potentially allowing lower, less toxic chemotherapy doses [36]. The combination of recombinant measles virus and the PARP inhibitor olaparib markedly enhanced cytotoxicity in PDAC models, inducing cell cycle arrest and caspase-dependent apoptosis with minimal off-target effects [37].

Advanced MeV constructs further improve targeting. A miRNA-detargeted strain significantly reduced tumor volumes and extended survival in mice [38]. A PSCA-retargeted MeV expressing PNP, combined with fludarabine, induced substantial tumor cell death, including in gemcitabine-resistant lines, highlighting utility in refractory disease [39]. Similarly, a cytosine deaminase-armed MeV with 5-fluorocytosine showed promise, benefiting from miR-148a detargeting and compatibility with existing gastrointestinal cancer regimens [40].

Each strategy presents distinct benefits: HDACis enhance tumor susceptibility to MeV epigenetically, gemcitabine facilitates clinical translation due to its established use, and prodrug-activating viruses offer precision cytotoxicity. Nonetheless, limitations remain: immune suppression, chemoresistance, and viral delivery hurdles. Future research must refine the balance between efficacy, immune activation, and delivery feasibility to fully realize MeV’s potential in pancreatic cancer treatment.

### 2.3. Newcastle Disease Virus

NDV, a negative-sense, single-stranded RNA virus from the Paramyxoviridae family, is primarily an avian pathogen but has emerged as a promising oncolytic agent due to its selective cytotoxicity toward cancer cells. NDV exists in three pathotypes: lentogenic (low virulence), mesogenic (moderate), and velogenic (high). Its genome encodes six structural proteins, including the fusion (F) and hemagglutinin-neuraminidase (HN) proteins. The F protein mediates viral entry via membrane fusion, while HN facilitates host cell binding through sialic acid recognition [41].

Lentogenic strains like LaSota and Hitchner-B1 are commonly used in cancer therapy due to their favorable safety profiles, though their limited cytolytic activity restricts standalone efficacy. To enhance their therapeutic potential, engineering strategies have been employed. One method introduces multiple arginine residues into the F protein’s cleavage site, making it more susceptible to tumor-associated proteases [42]. Another involves the L289A mutation, enhancing membrane fusion and syncytia formation, thereby increasing tumor cell killing. Additionally, NDV can be armed with TAAs, cytokines, tumor suppressors, or enzymes like MMP-8 to improve immune activation and intratumoral spread [43].

Preclinical studies support NDV’s tumor selectivity. R.J. Walter et al. showed NDV-LaSota (NDV-LS) was more cytotoxic to pancreatic cancer cells than healthy cells, although tumors from cystic fibrosis patients were less responsive, likely due to CFTR mutations resulting in membrane alterations or shifts in intracellular ion balance [44]. Another study comparing NDV-B1 and NDV-U in seven pancreatic cancer and four normal cell lines found NDV-B1 had higher selectivity and efficacy, while NDV-U required higher doses to achieve similar effects [45].

In vivo studies demonstrated that a single intravenous dose of NDV strain R75/98 significantly delayed pancreatic tumor progression and prevented recurrence in some mice for over three months. The therapeutic benefit stemmed from both direct tumor lysis and activation of natural killer cells, highlighting NDV’s dual role as an oncolytic and immune-stimulating agent [46].

Compared to other viruses like MeV, NDV offers superior safety and immunogenicity but requires genetic enhancement to match MeV’s cytolytic potency. Future strategies may involve combining NDV’s immune-stimulating capabilities with more aggressive oncolytic vectors for improved therapeutic outcomes in pancreatic cancer.

### 2.4. Reoviruses

Reoviruses, members of the Reoviridae family, are non-enveloped viruses with segmented double-stranded RNA genomes that typically cause mild or asymptomatic infections in humans. Their ten genome segments encode structural and non-structural proteins involved in replication, immune evasion, and host entry. Notably, the σ3 protein inhibits activation of protein kinase R, dampening the antiviral response, while σ1 mediates entry via receptors like sialic acid and junctional adhesion molecule-A [47].

Reovirus RNA is detected by pattern recognition receptors like RIG-I and MDA5, which activate IFN pathways to curb replication. However, reoviruses evade these defenses through mechanisms such as RNA masking and interference with immune signaling [48]. These immune evasion strategies are pivotal to their role as oncolytic agents.

Reovirus shows natural selectivity for cancer cells, particularly those with dysregulated Ras signaling. Ras activation enhances viral uncoating, replication, and apoptosis. However, KRAS mutations alone are not reliable predictors of response, as downstream signaling alterations and IFN pathway defects may be more decisive [49].

In preclinical PDAC models, reovirus induced selective tumor cell lysis without harming normal cells. Intratumoral injections led to tumor regression and systemic antitumor responses, including activity against liver metastases, an especially lethal complication in PDAC [50].

Despite strong preclinical data, clinical translation has faced hurdles. A phase 2 trial combining Pelareorep (Reolysin) with gemcitabine reported a 58% clinical benefit rate and 10.2-month median overall survival (OS), similar to existing therapies but with lower toxicity [51]. However, the study’s small size and lack of control arm limit conclusions. A meta-analysis found no significant OS improvement with reovirus-chemotherapy combinations and noted increased adverse events [52].

Checkpoint inhibitor combinations have also yielded modest results. A trial with Pelareorep and pembrolizumab in PDAC patients showed limited benefit (OS 6.3 months; 42% clinical benefit) [53], and adding Pelareorep to paclitaxel/carboplatin failed to enhance progression-free survival [54].

Compared to MeV or NDV, reovirus offers moderate oncolytic activity but excels in safety and systemic delivery. Its selectivity for Ras-driven and hypoxic tumors is promising [55], but inconsistent outcomes and a lack of predictive biomarkers remain obstacles. Future work should focus on patient stratification, identifying biomarkers like TRAIL receptors or CUG2 [56,57], and improving delivery and trial design to fully realize its potential in PDAC.

### 2.5. Herpes Simplex Virus

HSVs are enveloped, double-stranded DNA viruses from the Herpesviridae family. HSV-1 and HSV-2 are the two primary human pathogens, causing orofacial and genital infections, respectively [58]. The HSV genome encodes over 80 polypeptides, including structural components such as capsid and tegument proteins, and envelope glycoproteins like gD, which facilitate viral entry by binding to host cell receptors including nectin-1 and HVEM [59]. The viral gene expression program is orchestrated by immediate early proteins such as ICP0, ICP4, and ICP22.

A critical viral enzyme, thymidine kinase (TK), facilitates viral DNA replication by phosphorylating nucleosides. TK is also leveraged in antiviral therapy, as it activates prodrugs like ganciclovir. In oncolytic virotherapy, retaining TK allows for pharmacologic control of viral spread. Moreover, TK can serve as a suicide gene: when paired with ganciclovir, it enables selective destruction of HSV-infected cancer cells [60,61].

HSV is among the most promising oncolytic viruses. Its therapeutic variant, talimogene laherparepvec (T-VEC), engineered to express GM-CSF, is FDA-approved for melanoma treatment. While this approval underscores HSV’s clinical relevance, translating this success to pancreatic ductal adenocarcinoma (PDAC) remains challenging. Species-specific receptor usage complicates preclinical modeling, as HSV infects human cells via receptors like HVEM, which murine cells do not naturally express. To address this, HSV-susceptible murine PDAC lines (e.g., Pan02_HVEM) have been developed [62].

To improve tumor selectivity and immunogenicity, oncolytic HSVs (oHSVs) are frequently engineered with deletions in virulence genes such as ICP34.5, ICP6, and ICP47. ICP34.5 plays a key role in antagonizing the PKR-mediated antiviral pathway by reversing translational arrest through phosphatase recruitment [63]. Its deletion renders the virus attenuated in healthy cells with intact antiviral responses, while allowing preferential replication in cancer cells, which often have defective PKR signaling.

ICP6, the large subunit of ribonucleotide reductase encoded by UL39, enables replication in non-dividing cells. Deletion of ICP6 curtails viral proliferation in normal tissues but retains efficacy in rapidly dividing cancer cells due to their high nucleotide demand. This mutation underpins the rationale for combining oHSVs with chemotherapeutics like 5-FU, which can further increase cellular dependence on RNR activity and enhance viral efficacy [64]. ICP47 disrupts antigen presentation by blocking TAP-dependent peptide transport to MHC-I. Its removal restores antigen visibility and enhances CD8^+^ T cell responses [65]. This design choice transforms oHSV from a solely lytic agent into a platform for antitumor immunomodulation.

In addition to direct cytolysis, oHSVs induce immunogenic cell death (ICD), characterized by the release of DAMPs such as ATP, calreticulin, and HMGB1 [66]. These signals promote dendritic cell maturation and T cell priming, thereby enhancing systemic antitumor immunity. However, while ICD is crucial for turning “cold” tumors “hot,” its effectiveness in highly immunosuppressive tumors like PDAC is limited.

To bolster immunogenicity, oHSVs have been armed with immunomodulatory transgenes. GM-CSF remains the best-characterized example, enhancing dendritic cell recruitment and antigen presentation. Other modifications include IL-12, which promotes Th1 polarization [67], and IL-2, which stimulates T cell proliferation [68]. Despite these innovations, robust systemic immune activation remains elusive in PDAC, partly due to the tumor’s immunosuppressive microenvironment and the localized nature of viral delivery.

A major limitation of oHSV therapy in PDAC is the method of administration. Intratumoral injection ensures local viral presence but limits applicability for metastatic disease. Systemic administration is often hampered by rapid immune clearance. Additionally, cytokine-armed oHSVs carry the risk of inducing autoimmune-like effects if expression is not properly localized. Innovations such as tumor-specific promoters, viral coating strategies for circulation stability, and cell-based delivery systems are under investigation [69], but have yet to be fully realized in PDAC.

Several oHSV strains have been tailored for oncolytic use, though only a subset have been tested in PDAC models. These include hrR3 (ICP34.5-deleted), NV1020 (ICP34.5 and UL56-deleted), G207 (deleted ICP34.5 and ICP47), HF10 (UL43 and UL56-deleted), and NV1066 (ICP34.5-deleted, GFP-expressing). Each carries specific design features intended to balance safety with antitumor efficacy.

In preclinical PDAC models, oHSV has been shown to remodel the TME, reducing TAMs and enhancing lymphocytic infiltration. For instance, combining oHSV with immune checkpoint blockade (e.g., anti-CTLA-4 or anti-OX40) has produced synergistic effects and improved survival in murine models [62]. VG161, an engineered strain expressing immunostimulatory cytokines and a PD-L1-blocking peptide, has shown potent in vitro immunomodulatory effects, warranting further evaluation [70].

Compared to other oncolytic viruses like reovirus or adenovirus, HSV provides a larger genome for genetic manipulation, enabling complex arming strategies. While reovirus offers natural tumor selectivity through Ras pathway exploitation, and adenoviruses offer broad tropism and easy production, HSV’s ability to carry immunomodulatory genes gives it an edge in designing tailored antitumor responses, particularly if delivery and systemic efficacy challenges can be addressed.

A phase 1 clinical trial using HF10 in six PDAC patients showed encouraging trends. Although all patients developed HSV antibodies, which might have predicted rapid viral clearance, oHSV still spread efficiently through tumor tissue. One patient showed partial response, and four exceeded median survival for patients undergoing palliative surgery [71]. These results suggest that pre-existing immunity does not preclude therapeutic benefit, though efficacy remains variable.

Thus, HSV-based oncolytic virotherapy presents a versatile and promising approach for PDAC. However, its clinical translation demands improvements in systemic delivery, biomarker-based patient stratification, and combination strategies with immunotherapies. Compared to other OV platforms, HSV stands out for its engineering flexibility, though overcoming PDAC’s immune barriers remains the key challenge ahead.

### 2.6. Influenza Virus

Influenza A virus (IAV), from the Orthomyxoviridae family, is an enveloped, negative-sense, single-stranded RNA virus with a segmented genome. Among influenza types A–D, type A is most studied in cancer therapy due to its broad host range and high mutability. It is subtyped based on surface glycoproteins haemagglutinin (HA) and neuraminidase (NA). HA enables viral entry by binding to sialic acid on host cells, while NA cleaves sialic acid to facilitate viral release [72].

IAV also encodes proteins essential for replication and immune evasion. These include the RNA polymerase complex, M1 for virion assembly, and M2 for uncoating. Among the nonstructural proteins, NS1 is a key immune antagonist that inhibits IFN signaling and activation of PKR, while NS2 facilitates nuclear export of viral ribonucleoproteins [73]. In cancer therapy, IAV exhibits a biphasic effect on apoptosis. NS1 initially blocks apoptosis to support replication, but later, proteins like PB1-F2 and NP promote cell death, making it effective against apoptosis-defective tumors like PDAC [74].

Tumor selectivity can be enhanced through genetic modifications. Altering the HA cleavage site to require elastase, a protease abundant in PDAC, enables tumor-specific activation. Deleting NS1 confines replication to IFN-deficient cancer cells and boosts immune responses [75,76].

Compared to chemotherapies, IAV induces stronger apoptosis in PDAC models. In vitro, it outperforms gemcitabine and cisplatin [77]. Highly pathogenic strains like H5N1 show superior replication and cytokine release in PDAC cells over H1N1 and H7N2 [78]. A truncated-NS1 H7N3 variant selectively replicated in tumor cells and significantly reduced tumor burden in vivo [79].

IAV’s IFN sensitivity and pro-apoptotic action offer a unique therapeutic angle. However, its systemic immunogenicity may limit repeated use, necessitating optimized delivery and immune modulation strategies for PDAC.

### 2.7. Poxvirus

VACV, a double-stranded DNA virus from the Poxviridae family, is a strong candidate for oncolytic virotherapy. Historically used in smallpox vaccination, VACV replicates rapidly in the cytoplasm, even under hypoxia, and its large genome supports extensive genetic engineering. Importantly, VACV does not integrate into host DNA [80].

Tumor selectivity is enhanced by deleting viral genes like thymidine kinase (TK) and ribonucleotide reductase (RNR), both overexpressed in cancer cells [81]. Additional attenuation involves removing virulence genes such as F13L [82] and immune evasion genes like E5R [83], improving immune recognition of infected tumor cells. JX-594 (Pexa-Vec), a TK-deleted VACV expressing GM-CSF, has reached phase 3 trials in hepatocellular carcinoma, showing the platform’s clinical viability [84].

In PDAC, VACV has demonstrated encouraging results. A modified strain expressing a human endostatin–angiostatin fusion gene overcomes limitations of conventional anti-angiogenic therapy by enabling sustained, tumor-selective inhibition of angiogenesis [85]. VACVs engineered to express tumor antigens CEA and MUC-1 induced immune responses correlated with survival in PDAC patients, representing a cancer vaccine approach rather than classical oncolytic virotherapy [86]. Another variant, VVL-21, expressing IL-21, enhanced antitumor immunity and survival [87]. A survivin-targeted strain combined with gemcitabine improved outcomes in survivin-overexpressing PDAC, a group with poor prognosis and chemoresistance [88].

VACV’s engineering flexibility, systemic delivery potential, and antitumor efficacy make it a promising platform for treating PDAC.

### 2.8. Parvoviridae

H-1 parvovirus (H-1PV), a non-pathogenic rat Protoparvovirus with a single-stranded DNA genome, shows strong oncolytic activity and does not infect humans. Its antitumor effects are mediated by immunogenic cell death and the release of DAMPs like calreticulin, ATP, and HMGB1, which activate antitumor immunity [89]. Tumor selectivity is driven by cancer-specific features such as defective antiviral responses and disrupted cell cycle control.

In PDAC, H-1PV has shown efficacy alone and in combination therapies. H-1PV synergizes with gemcitabine to enhance efficacy, reduce drug dose, and prevent metastasis in vivo [90]. H-1PV-mediated chemokine delivery boosted immune cell infiltration, especially NK cells and monocytes, leading to potent antitumor effects in preclinical models [91]. Co-treatment with valproic acid, a histone deacetylase inhibitor, further enhanced therapeutic outcomes [92].

A phase 2 trial of ParvOryx, an H-1PV-based therapy, in metastatic PDAC patients showed good safety, with two patients achieving partial remission and extended survival [93]. Compared to more complex viruses, H-1PV offers a selective, minimalistic platform that could be enhanced by combining with immune checkpoint inhibitors.

**Figure 2:**
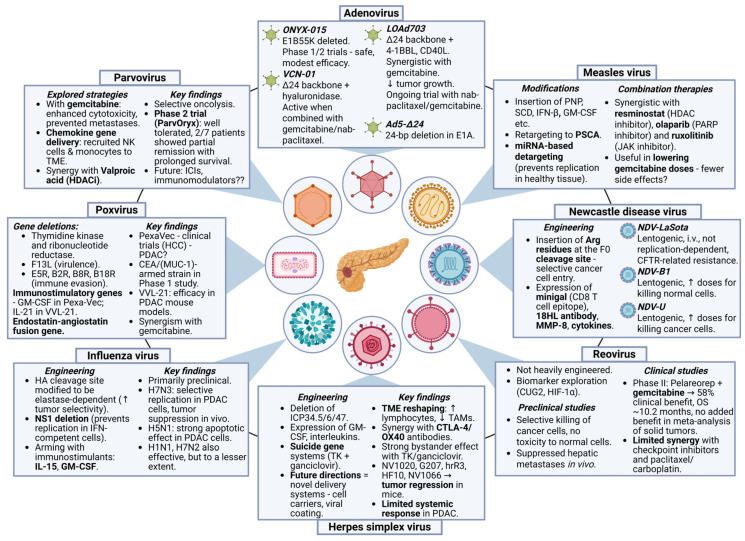
Comparative schematic presentation of oncolytic viruses involved in PDAC, highlighting their specific characteristics. The ↑ arrow suggests the increase of the parameters, while ↓ arrow suggests the decrease of the parameters.

A schematic representation of the oncolytic viruses involved in PDAC with their characteristics is presented in Figure 2. Table 1 summarizes selected clinical trials of OVs in PDAC.

### 2.9. Combination Strategies: A Brief Perspective

Although combination strategies with OVs are discussed throughout this review, it is worth emphasizing their central importance. OVs are increasingly evaluated alongside chemo- and immunotherapy, with the rationale of enhancing immunogenic cell death and overcoming resistance mechanisms. While early results are encouraging, optimizing scheduling, dosing, and patient selection remains critical for maximizing efficacy. Combination with chemotherapies is supported by a growing body of preclinical and early clinical evidence, as highlighted throughout this review. The rationale for combining OVs with chemotherapy in PDAC is to leverage the direct cytolytic effects of OVs and their ability to stimulate anti-tumor immunity, while chemotherapy can debulk tumors, modulate the TME, and enhance viral replication and immune cell infiltration. Notably, OVs armed with immune-stimulatory cytokines further potentiate effector lymphocyte cytotoxicity and modulate fibroblast populations, amplifying antitumor immunity when combined with chemotherapy [94,95].

Another combination approach emphasized in this review is that of OVs with immunotherapies. Mechanistically, OVs can convert immunologically “cold” TMEs into “hot” states, thereby enhancing the efficacy of immune checkpoint inhibitors [96].

Perhaps the most promising avenue is combining OVs with adoptive cellular therapies. Chimeric antigen receptor (CAR)-T cells, patient-derived lymphocytes engineered to express CARs, bypass MHC-TCR–dependent antigen recognition. While transformative in hematologic malignancies, CAR-T therapy faces major hurdles in PDAC, including an immunosuppressive TME, dense stroma restricting infiltration, and antigen heterogeneity. OVs may help overcome these barriers by remodeling suppressive niches, promoting epitope spread, and providing direct tumor lysis [97]. Preclinical studies demonstrate that OVs can synergize with mesothelin-targeted CAR-T cells: oncolytic HSV delivering mesothelin or adenoviruses expressing TNF-α and IL-2 enhance CAR-T activation, increase T cell infiltration, reprogram macrophages and dendritic cells, and prevent metastasis, collectively amplifying antitumor efficacy in PDAC models [98,99]. These findings highlight OV–CAR-T combinations as a promising strategy to surmount the immunosuppressive landscape of pancreatic tumors.

## 3. Cancer Vaccines in PDAC

Vaccination has been a major success in preventing infectious diseases, but applying it to treat cancer remains difficult. The TME hinders immune cell infiltration, antigen recognition, and tumor cell lysis. Cancer vaccines aim to stimulate immune cells to recognize tumor-associated and TSAs on malignant cells [100].

Despite significant research, most cancer vaccines have not substantially improved survival or quality of life, likely due to poor antigen selection, weak formulations, and trial design. Key criteria have emerged for effective vaccine development [101]:

- Efficient antigen presentation by dendritic cells (DCs), with synthetic long peptides (SLPs, >20 amino acids) offering more robust CD4^+^ and CD8^+^ T-cell activation [102].
- Optimized delivery systems.
- Use of strong adjuvants.
- Combination with other therapies.

To date, Provenge^®^ (Sipuleucel-T) is the only FDA-approved cancer vaccine for metastatic castration-resistant prostate cancer [103].

Key vaccination strategies include direct antigen delivery, tumor cells, or autologous antigen-presenting cells. The following sections will review cell-, bacterial-, peptide-, and nucleic acid-based vaccines in pancreatic cancer.

### 3.1. Whole-Cell Vaccines

This vaccine strategy employs whole irradiated tumor cells or DCs engineered to present specific TAAs/TSAs. Irradiated tumor cells express a broad array of antigens, promoting comprehensive immune activation. In contrast, DCs can be loaded ex vivo with defined antigens to elicit a targeted immune response.

Autologous cells, derived from the same patient, offer the most personalized antigen source, reflecting the individual’s unique tumor profile and minimizing immune mismatch. However, in PDAC, this approach faces practical hurdles. Few patients present with resectable tumors suitable for harvesting, and pancreatic biopsies are technically complex, often requiring advanced imaging or endoscopy.

Allogeneic vaccines, derived from donor cells or established PDAC cell lines, provide a scalable and consistent alternative. They can be manufactured in bulk and administered widely. Yet, this strategy is limited by PDAC’s genetic and antigenic heterogeneity, potentially overlooking unique neoantigens in individual tumors.

Thus, while allogeneic approaches are more feasible, balancing manufacturing practicality with immunologic specificity remains a major challenge in developing effective cell-based vaccines for PDAC.

#### 3.1.1. GVAX

GVAX is a whole-cell-based vaccine developed for pancreatic cancer, using two irradiated allogeneic pancreatic cancer cell lines engineered to secrete GM-CSF. These cells serve as both antigen sources and immune adjuvants. GM-CSF promotes recruitment and activation of antigen-presenting cells (APCs), enhancing MHC I/II presentation and subsequent activation of CD4^+^ and CD8^+^ T cells. This broad antigen presentation is advantageous in PDAC, where tumor-specific antigens are poorly defined. A phase 1 trial in 14 patients with resected stage II/III PDAC showed GVAX was well tolerated, with delayed-type hypersensitivity and prolonged disease-free intervals in three patients (25–30 months) [104].

In a phase 2 study of 50 advanced PDAC patients, GVAX ± low-dose cyclophosphamide (Cy, to inhibit Tregs) showed mesothelin-specific T-cell responses, but no significant survival benefit (69 vs. 130 days), likely due to cohort imbalances [105]. As adjuvant therapy with 5-FU and radiation post-resection, GVAX improved disease-free (17.3 months) and overall survival (24.8 months), with mesothelin-specific responses linked to better outcomes [106]. GVAX combined with ipilimumab (anti-CTLA-4) extended median OS (5.7 vs. 3.6 months) in a phase 1b trial. However, only patients with OS > 3.4 months showed strong T-cell responses, indicating variability in immunogenicity [107]. Following FOLFIRINOX, GVAX/ipilimumab underperformed compared to continued chemotherapy (9.38 vs. 14.7 months), despite immune activation [108].

GVAX’s impact is often limited by the immunosuppressive TME, which impedes T-cell infiltration. Still, a phase 2 neoadjuvant trial showed intratumoral lymphoid aggregate formation in 33/39 resected tumors, absent in historical controls. Patients also exhibited improved Teffector/Treg ratios and mesothelin-specific responses, especially with Cy co-treatment, successfully converting a “non-immunogenic” neoplasm into an “immunogenic” neoplasm [109]. Similarly, in a neoadjuvant platform trial for resectable PDAC, GVAX vaccination combined with PD-1 blockade and the CD137 agonist urelumab significantly increased intratumoral CD8^+^ CD137^+^ T cells, reflecting enhanced cytotoxic activation. All regimens were well-tolerated, and the triple combination showed numerically superior disease-free and overall survival compared to GVAX alone or with PD-1 blockade, suggesting that rational immunotherapy combinations can potentiate antitumor immunity even in highly suppressive pancreatic tumors [110].

To enhance efficacy, GVAX was combined with CRS-207 (Listeria monocytogenes expressing mesothelin). A phase 2 trial showed modest OS improvement [111], but a larger phase 2b trial found no survival difference versus CRS-207 alone or chemotherapy [112]. Adding nivolumab (anti–PD-1) also failed to improve OS, though TME modulation was seen in long-term survivors [113].

Overall, GVAX shows immunologic activity and a good safety profile, but limited and inconsistent clinical benefit. Overcoming tumor heterogeneity, immune evasion, and TME barriers may require more personalized vaccine strategies and optimized combinations. Ongoing trials (e.g., NCT03190265, NCT03767582, NCT03153410, NCT03006302, NCT02451982, NCT03161379, NCT01896869) aim to clarify its role in PDAC.

#### 3.1.2. Dendritic Cells-Based Vaccines

Dendritic cells (DCs), first distinguished from macrophages by Ralph Steinman [114], are central to initiating adaptive immunity. Located in peripheral tissues, DCs capture and present antigens via MHC class I and II molecules, priming naïve CD4^+^ and CD8^+^ T-cells in lymphoid organs. In pancreatic cancer, tumor cells and the immunosuppressive TME impair DC recruitment, antigen presentation, and T-cell activation, limiting their antitumor efficacy [115].

DCs have been exploited in cancer vaccines. Ex vivo–generated DCs are pulsed with TAAs, matured, and reinfused to elicit cytotoxic T-cell responses [116]. Although some results are promising, clinical responses remain inconsistent. A strong erythema reaction at the injection site may indicate vaccine activity.

In murine models, DCs pulsed with mesothelioma-associated antigens (also present in PDAC) delayed tumor growth prophylactically but were ineffective against established tumors. However, combining this vaccine with FGK45 (a CD40 agonist) led to tumor regression and complete response in 56% of mice, alongside reduced T cell exhaustion and increased PD-L1 expression [117].

In a phase 1/2 trial, 38 PDAC patients post-resection received an autologous DC vaccine pulsed with mesothelioma lysate after chemotherapy. Two-year recurrence-free survival reached 64%, supporting its adjuvant potential [118]. Preclinical studies using Panc02 cell lysate–pulsed DCs also showed improved survival [119]. A phase 1 trial of a Mucin-1 (MUC-1) peptide-pulsed DC vaccine in seven advanced PDAC patients demonstrated safety, though no clinical responses were observed [120]. A Phase 1b trial is assessing personalized neoantigen-pulsed DC vaccines with chemotherapy and nivolumab in resectable PDAC [121].

A combination of gemcitabine and S-1 with DC vaccination in 49 unresectable PDAC patients yielded a median OS of 508 days, surpassing DC vaccine/GEM (360 days) and DC vaccine/S-1 (168 days). Two patients achieved complete remission; four survived >500 days [122].

A WT1/MUC-1–pulsed DC vaccine used as adjuvant therapy with gemcitabine/S-1 in resected PDAC patients showed a 3-year OS of 77.8%, higher than JASPAC-01’s 59.7% for S-1 alone [123,124]. However, 5-year OS dropped to 19.4%, with RFS at 23.3%, suggesting early benefit but poor long-term control.

In metastatic PDAC, WT1-DC vaccination plus gemcitabine yielded a median OS of 717 days in patients with strong delayed-type hypersensitivity reactions [125]. Another trial showed limited benefit, with efficacy restricted to patients without liver metastases [126]. Low IL-6/IL-8 levels correlated with improved outcomes, suggesting potential biomarkers [127].

Vaccell, a DC-based vaccine matured with streptococcal adjuvants, combined with chemotherapy, achieved a median OS of 16.5 months in 255 inoperable PDAC patients, though smaller prospective studies showed modest efficacy [128].

DCs pulsed with hTERT, CEA, and survivin combined with poly-ICLC (a TLR agonist) induced stable disease in 4 of 8 advanced PDAC patients. Median OS was 7.7 months, outperforming second-line treatments like those in NAPOLI-1 (6.1 vs. 4.2 months) [129,130].

Overall, DC vaccines may provide additive benefits, especially when paired with chemotherapy or immunomodulators. However, variability in outcomes, antigen targets, and study designs hampers broad clinical adoption. Larger, controlled trials are needed to refine vaccine formulations, identify biomarkers, and define optimal patient subsets.

#### 3.1.3. Algenpantucel-L

Algenpantucel-L is a whole-cell vaccine derived from two irradiated allogeneic PDAC cell lines engineered to express αGT. The α-Gal epitopes on these cells trigger immune attack, simultaneously exposing other TAAs to the immune system, promoting broader antitumor recognition [131]. In a phase 2 trial combined with adjuvant gemcitabine, 5-FU, and radiation, the vaccine was safe and well-tolerated but showed no clinical benefit [132]. Similarly, a phase 3 trial in patients with borderline resectable or unresectable PDAC found that adding Algenpantucel-L to standard neoadjuvant chemotherapy (FOLFIRINOX or gemcitabine/nab-paclitaxel) followed by chemoradiation did not improve outcomes, confirming its limited therapeutic value in this setting [133].

#### 3.1.4. Bacterial-Based Vaccines

Listeria monocytogenes (Lm) is a Gram-positive bacterium known for causing gastroenteritis in healthy individuals but can lead to severe systemic infections in vulnerable populations [134]. Attenuated Lm strains have been investigated as vectors for delivering TAAs in cancer immunotherapy, including PDAC. Genetic deletions of virulence factors like internalin B and actA reduce pathogenicity while maintaining immunogenicity [135].

Clinical trials have tested Lm-based vaccines such as ANZ-100 (a live-attenuated double-deleted LADD strain) and CRS-207 (engineered to express mesothelin). These agents were generally well tolerated, causing only mild bacteremia, and demonstrated potential when used in combination therapies, particularly with GVAX. However, CRS-207 monotherapy showed limited efficacy, highlighting the importance of combination strategies [136].

Salmonella species, Gram-negative bacteria known for their natural tumor-targeting abilities, have also been explored for cancer therapy. Attenuated strains are created by deleting metabolic and virulence genes such as aroA, guaBA, hrtA, and ssaV, reducing toxicity while retaining tumor-colonizing capacity [137]. These bacteria preferentially localize in hypoxic tumor regions, making them effective delivery systems for immunostimulatory molecules [138].

A Salmonella-based therapy targeting IDO and expressing the PEGPH20 hyaluronidase showed strong antitumor effects in PDAC models by enhancing PMN infiltration and inducing tumor regression [139]. Another promising Salmonella-based agent is VXM01, an oral vaccine using Salmonella typhi to express VEGFR2, a key mediator of tumor angiogenesis. In a phase 1 trial, VXM01 was safe in advanced PDAC patients and induced biomarkers consistent with anti-angiogenic activity [140]. Future trials aim to evaluate its combination with gemcitabine in stage 4 PDAC, potentially advancing to phase 2 [141].

Comparing the two platforms, Listeria is adept at activating cytotoxic T cells via MHC class I pathways but carries higher safety risks. Salmonella offers safer, scalable, and tumor-targeting delivery with oral dosing advantages. Despite early promise, both platforms face obstacles, including tumor heterogeneity and immunosuppressive microenvironments. Thus, their greatest therapeutic value may lie in synergistic combination regimens rather than as monotherapies. Continued clinical trials are essential to clarify their roles in PDAC treatment.

### 3.2. Other Types of Vaccines

#### 3.2.1. Peptide-Based Strategies

Active vaccination strategies aim to stimulate T-cell–mediated antitumor responses by delivering TAAs or TSAs, which are processed by antigen-presenting cells and presented to T-cells. This approach has been explored across various cancers [142].

One example is KIF20A-66, a peptide derived from the kinesin family member KIF20A, overexpressed in PDAC and associated with tumor aggressiveness [143]. In a phase I trial with 29 advanced PDAC patients, KIF20A-66 vaccination led to stable disease in 21 and tumor shrinkage in 8, including one complete response. Median OS was 142 days versus 83 days in controls, suggesting modest benefit [144].

Telomerase, reactivated in cancer but silent in normal cells, is another immunotherapy target and a reliable PDAC biomarker [145]. GV1001, a telomerase-derived peptide vaccine given with GM-CSF, induced immune responses in 24 of 38 patients, with a one-year survival of 25% and median OS of 8.6 months in the intermediate-dose group [146]. However, the large phase 3 TeloVac trial (*n* = 1062) showed no survival benefit when GV1001 was added to chemotherapy [147]. These findings underscore the challenge of converting vaccine-induced immune responses into durable clinical outcomes. Future strategies may require more potent hTERT-targeted immunotherapies or combination approaches.

#### 3.2.2. DNA-Based Strategies

DNA vaccines offer a relatively simple and scalable immunotherapy approach, using bacterial plasmids that act as ligands for Toll-like receptors (TLRs) on immune cells, thereby activating innate immunity [148]. Unlike protein- or peptide-based vaccines, DNA vaccines also promote endogenous antigen presentation and are more stable, although their clinical translation has been limited by inconsistent immunogenicity in humans.

One promising target is α-enolase (ENO1), a glycolytic enzyme overexpressed in PDAC that also facilitates tumor progression and immune evasion. A DNA vaccine against ENO1 induced complement-mediated cytotoxicity and slowed tumor growth in mice while sparing normal tissue [149]. Combining gemcitabine enhanced immune responses to ENO1 and G3P, highlighting the synergy between chemotherapy and immunization [150]. Cancer-associated fibroblasts, particularly those expressing fibroblast activation protein α (FAPα), help shape the immunosuppressive TME in PDAC. A DNA vaccine targeting both FAPα and survivin, combined with gemcitabine, significantly suppressed tumor growth and reprogrammed the TME in murine models [151].

Another key antigen is MUC1, aberrantly glycosylated in over 90% of PDAC cases [152]. A DNA vaccine encoding MUC1’s VNTR domain, such as pVAX1-MUC1-VNTR6, showed potent antitumor activity in vitro and in vivo [153].

These preclinical results support the potential of DNA vaccines, especially when combined with cytotoxic agents, though clinical validation remains essential.

#### 3.2.3. RNA-Based Strategies

The unprecedented success of COVID-19 mRNA vaccines has reignited interest in leveraging this platform for oncology. mRNA vaccines offer rapid, scalable production, strong immunogenicity, and the flexibility to encode multiple antigens, advantages that surpass many DNA- and peptide-based approaches. Advances in delivery systems further reinforce their potential as transformative tools in cancer immunotherapy [154,155].

In PDAC, immunoinformatics-driven design has enabled the generation of promising multi-epitope constructs. For instance, an mRNA vaccine targeting S100 family proteins, key activators of RAGE signaling, demonstrated predicted stability, optimized expression, and strong TLR2/4 affinity, supporting its ability to elicit both humoral and cellular immunity, although clinical validation remains pending [156]. Complementing these efforts, integrative analyses of TCGA and GTEx datasets identified immune- and oxidative stress–related gene signatures with prognostic significance. A four-gene model (ERAP2, MET, CXCL9, AGT) not only improved outcome prediction but also highlighted potential mRNA vaccine targets, linking tumor mutational burden, immune infiltration, and metabolic pathways to translational vaccine development; however, these genes remain untested in preclinical or clinical PDAC models [157].

At the clinical level, the individualized neoantigen vaccine autogene cevumeran, administered alongside atezolizumab and mFOLFIRINOX in PDAC patients, induced long-lived CD8^+^ T-cell clones with memory-like, cytotoxic phenotypes, translating into markedly prolonged recurrence-free survival in responders. These findings underscore the ability of mRNA neoantigen vaccines to overcome a central challenge in cancer vaccination, generating durable, functional T-cell immunity, though results are preliminary and derived from early-phase, small-cohort studies, highlighting the need for validation in larger, randomized trials [158].

**Figure 3:**
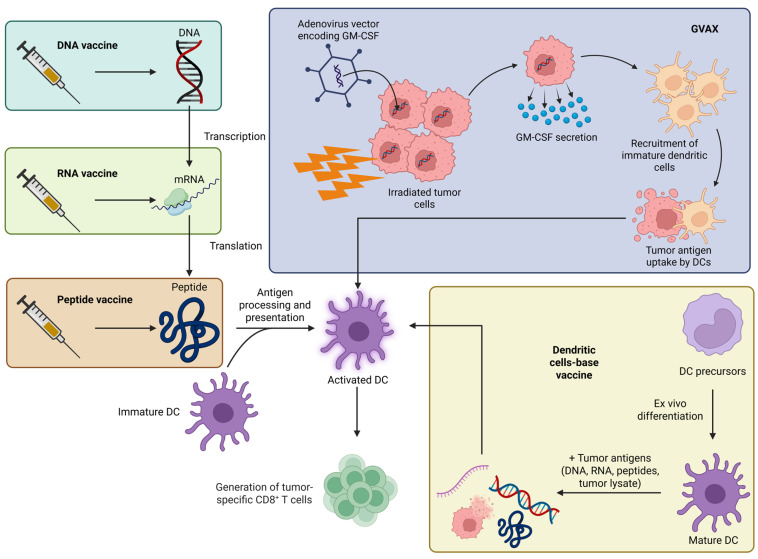
Classification of cancer vaccines in PDAC and representation of their mechanism of action.

Collectively, these studies illustrate that mRNA vaccines hold significant promise for PDAC by enabling rational antigen selection, integrating molecular signatures into precision immunotherapy, and inducing long-lived antitumor immunity. Nevertheless, bridging the gap between computational design, preclinical validation, and clinical translation remains a critical challenge for the field. Figure 3 schematically presents the classification of cancer vaccines in PDAC and their mechanism of action. Table 2 summarizes key clinical trials of therapeutic vaccines in PDAC.

## 4. Perspective and Conclusions

This review departs from conventional assessments of oncolytic virotherapy and cancer vaccines in PDAC by offering a mechanistically grounded and clinically oriented perspective on why these modalities have underperformed—and how they might be meaningfully repurposed. Rather than reiterating existing clinical trial outcomes or broadly cataloging therapeutic agents, this review provides a critical discussion of the convergence between tumor biology and therapeutic design. It emphasizes the centrality of the TME as both a physical and immunological barrier, and explores how these therapies must evolve to overcome that complexity.

Despite encouraging results in other cancers, the clinical impact of oncolytic viruses and antitumor vaccines in PDAC has remained limited. This shortfall reflects not just the biological complexity of PDAC, but the inadequacy of monotherapies in addressing its multifaceted barriers, namely poor immunogenicity, a dense desmoplastic stroma, restricted viral penetration, and profound immune evasion.

The pancreatic TME acts as both a physical and immunological fortress. Effective immunotherapy must do more than deliver antigens or lyse tumor cells—it must penetrate stromal barriers, modulate suppressive immune networks, and promote durable immune memory. This requires multi-modal strategies that integrate oncolytic virotherapy and vaccination platforms with agents that target the stroma, vasculature, or immune checkpoints.

The path forward lies not in abandoning these approaches, but in re-engineering them. Rational combinations, such as oncolytic viruses engineered to express immunostimulatory cytokines, paired with checkpoint inhibitors or stromal modulators, are showing promise in preclinical studies. Similarly, vaccines based on personalized neoantigens or delivered via advanced platforms (e.g., nanoparticles, bacterial vectors) may circumvent prior limitations.

Rather than a failure, the current clinical plateau may represent a pivotal turning point. Lessons learned from early-phase trials highlight the importance of patient stratification, optimized delivery systems, and adaptive trial designs. Emerging technologies in synthetic biology and intratumoral immunomodulation offer powerful tools to reframe how we deploy these therapies.

Future progress will depend on integrating these platforms into well-designed combination regimens tailored to individual tumor biology. Success will also require deeper insights into resistance mechanisms, alongside continued investment in translational and multidisciplinary research.

Ultimately, oncolytic viruses and cancer vaccines should not be seen as obsolete tools, but as evolving components of a more sophisticated immunotherapeutic arsenal. In the landscape of pancreatic cancer, they represent not a dead end, but an unfinished chapter—one still rich with potential.

## Figures and Tables

**Table 1:** Representative clinical trials of oncolytic viruses in pancreatic cancer; not all trials are listed. The ↑ arrow suggests the increase of the parameters.

Viral Vector	Payload	N	Phase	Combination Therapy	Key Outcomes	Ref.
Adenovirus	-	21	I/II	Gemcitabine	Well tolerated; limited efficacy	[21]
Adenovirus	-	23	I	-	Well tolerated; limited efficacy	[22]
Adenovirus	Trimerized, membrane-bound CD40L + 4-1BBL	21	I/II	Nab-paclitaxel + gemcitabine	↑ CD8^+^ effector memory cell infiltration in 94% of patients	[23]
Adenovirus	Hyaluronidase	26	I	Nab-paclitaxel + gemcitabine	ORR = 50%, ↑ immune biomarkers	[24]
Reovirus	-	34	II	Gemcitabine	Median OS = 10.2 months; ↑ PD-L1	[49]
Reovirus	-	12	II	Pembrolizumab	Modest efficacy; inflamed TME	[51]
Reovirus	-	73	II	Carboplatin/paclitaxel	Safe, no PFS benefit regardless of KRAS status	[52]
HSV	-	6	I	-	Moderate efficacy in 4 patients, progression in 2	[62]
Vaccinia	CEA + MUC1, costimulatory molecules (B7.1, ICAM-1, LFA-3)	10	I	-	Median OS = 6.3 months; ↑ OS in immune responders	[84]
Parvovirus	-	7	II	Gemcitabine	↑ survival in responders	[91]

**Table 2:** Representative clinical trials of antitumor vaccines in pancreatic cancer; not all trials are listed. The ↑ arrow suggests the increase of the parameters, while ↓ arrow suggests the decrease of the parameters.

Vaccine Platform	Antigen/Payload	N	Phase	Combination Therapy	Key Outcomes	Ref.
Tumor cell (GVAX)	GM-CSF	14	I	Adjuvant 5-FU-based chemoradiation in 12/14 patients	DTH induced in 3/14 patients; all remained disease-free ≥ 25 months	[102]
Tumor cell (GVAX)	GM-CSF	50	I/II	-/Cy	OS = 2.3 mo (alone), 4.3 mo (+Cy); ↑ Mesothelin-specific CD8^+^ T cells with Cy	[103]
Tumor cell (GVAX)	GM-CSF	60	II	Adjuvant 5-FU-based chemoradiation	DFS = 17.3 mo; OS = 24.8 mo	[104]
Tumor cell (GVAX)	GM-CSF	30	I/II	Ipilimumab	↑ OS in combination therapy arm	[105]
Tumor cell (GVAX)	GM-CSF	82	II	Ipilimumab	Immune modulation observed; no OS benefit	[106]
Tumor cell (GVAX)	GM-CSF	40	Ib/II	Pembrolizumab	Numerically improved survival in Arm C; ↑ immune infiltration	[108]
Tumor cell (GVAX) + bacterial vector (CRS-207)	GM-CSF (GVAX), mesothelin (CRS-207)	90	II	Cy	↑ OS in combination arm vs. GVAX alone	[109]
Tumor cell (GVAX) + bacterial vector (CRS-207)	GM-CSF (GVAX), mesothelin (CRS-207)	303	IIb	Arm A: Cy/GVAX + CRS-207; Arm B: CRS-207 alone; Arm C: single-agent chemotherapy	No OS benefit for Cy/GVAX + CRS-207 vs. chemotherapy	[110]
Tumor cell (GVAX) + bacterial vector (CRS-207)	GM-CSF (GVAX), mesothelin (CRS-207)	93	II	Arm A: Cy/GVAX + CRS-207 + nivolumab; Arm B: Cy/GVAX + CRS-207	No OS benefit; ↑ CD8^+^ in long-term survivors	[111]
Dendritic cell	Tumor antigens	40	II	Gemcitabine	2-year DFS 38%; 2-year OS 55%	[116]
Dendritic cell	MUC1	8	I	-	Evidence of immunogenicity; occasional clinical benefit	[118]
Dendritic cell	Tumor antigens	49	I/II	Gemcitabine and/or S-1	Signals of clinical benefit; ↑ survival with lymphokine-activated killer cells; immunogenic	[120]
Dendritic cell	WT1 peptide + MUC1	10	I/IIa	-	2-year DFS 40%; 2-year OS 60%	[121]
Dendritic cell	WT1-specific MHC I/II epitopes	10	I/II	Gemcitabine, S-1 or both	Moderate efficacy; 1 partial response, 3 stable disease, 6 progressive disease	[123]
Dendritic cell	WT1 peptide	10	I	Gemcitabine	3 DTH+ patients had disease control; ↓ survival with liver metastases/high inflammatory markers	[124]
Dendritic cell	hTERT, CEA, survivin	12	I	Poly-ICLC (TLR3-agonist)	OS = 7.7 mo; antigen-specific T-cell responses in some patients	[127]
Tumor cell (Algenpantucel-L)	α(1,3)-galactosyltransferase	70	II	Adjuvant gemcitabine and 5-FU-based CRT	12-mo DFS: 62%; 12-mo OS: 86%	[130]
Tumor cell (Algenpantucel-L)	α(1,3)-galactosyltransferase	303	III	Neoadjuvant chemotherapy (FOLFIRINOX or gemcitabine/nab-paclitaxel) and chemoradiation	Addition of Algenpantucel-L did not improve OS or PFS	[131]
Bacterial vector (ANZ-100)	-	9	I	-	Well tolerated; immune activation	[134]
Bacterial vector (CRS-207	Mesothelin	17	I	-	Well tolerated; immune activation; 37% ≥ 15 mo survival	
Bacterial vector (VXM01)	VEGFR2	45	I	Gemcitabine	↑ VEGFR2-specific T cell responses; ↓ in tumor perfusion at day 38	[138]
Peptide	KIF20A-66	29	I/II	-	72% disease control rate; OS = 142 days; PFS = 56 days; ↑ survival compared to historical cohorts	[142]
Peptide (GV1001)	hTERT	48	I/II	GM-CSF	OS = 8.6 mo in intermediate-dose group, immune response correlated with prolonged survival	[144]
Peptide (GV1001)	hTERT	1062	III	Gemcitabine + capecitabine	No improvement in OS	[145]
mRNA (autogene cevumeran)	Individualized neoantigens	16	I	Atezolizumab, mFOLFIRINOX	↑ OS in responders; vaccine induced CD8^+^ T-cells in 8/16 patients	[156]
